# Brain fog in long COVID limits function and health status, independently of hospital severity and preexisting conditions

**DOI:** 10.3389/fneur.2023.1150096

**Published:** 2023-05-11

**Authors:** Anna S. Nordvig, Mangala Rajan, Jennifer D. Lau, Justin R. Kingery, Meem Mahmud, Gloria C. Chiang, Mony J. De Leon, Parag Goyal

**Affiliations:** ^1^Department of Neurology, Weill Cornell Medicine, New York, NY, United States; ^2^Division of General Internal Medicine, Department of Medicine, Weill Cornell Medicine, New York, NY, United States; ^3^Division of General Internal Medicine, University of Louisville School of Medicine, Louisville, KY, United States; ^4^Department of Radiology, Weill Cornell Medicine, New York, NY, United States; ^5^Division of Cardiology, Department of Medicine, Weill Cornell Medicine, New York, NY, United States

**Keywords:** brain fog, longCovid, COVID-19, cognitive dysfunction, long COVID disability, long COVID risk factors, post-acute sequelae of COVID-19

## Abstract

**Importance:**

The U.S. government has named post-acute sequelae of COVID-19 (longCOVID) as influential on disability rates. We previously showed that COVID-19 carries a medical/functional burden at 1  year, and that age and other risk factors of severe COVID-19 were not associated with increased longCOVID risk. Long-term longCOVID brain fog (BF) prevalence, risk factors and associated medical/functional factors are poorly understood, especially after mild SARS-CoV-2 infection.

**Methods:**

A retrospective observational cohort study was conducted at an urban tertiary-care hospital. Of 1,032 acute COVID-19 survivors from March 3–May 15, 2020, 633 were called, 530 responded (59.2 ± 16.3  years, 44.5% female, 51.5% non-White) about BF prevalence, other longCOVID, post-acute ED/hospital utilization, perceived health/social network, effort tolerance, disability.

**Results:**

At approximately 1-year, 31.9% (*n* = 169) experienced BF. Acute COVID-19 severity, age, and premorbid cardiopulmonary comorbidities did not differ between those with/without BF at 1  year. Patients with respiratory longCOVID had 54% higher risk of BF than those without respiratory longCOVID. BF associated with sleep disturbance (63% with BF vs.29% without BF, *p* < 0.0001), shortness of breath (46% vs.18%, *p* < 0.0001), weakness (49% vs.22%, *p* < 0.0001), dysosmia/dysgeusia (12% vs.5%, *p* < 0.004), activity limitations (*p* < 0.001), disability/leave (11% vs.3%, *p* < 0.0001), worsened perceived health since acute COVID-19 (66% vs.30%, *p* < 0.001) and social isolation (40% vs.29%, *p* < 0.02), despite no differences in premorbid comorbidities and age.

**Conclusions and relevance:**

A year after COVID-19 infection, BF persists in a third of patients. COVID-19 severity is not a predictive risk factor. BF associates with other longCOVID and independently associates with persistent debility.

## Introduction

Over 1 million adults endured coronavirus disease 2019 (COVID-19) in greater NYC; early estimates suggested up to 10% (1) suffered clinical Post-Acute Sequelae of COVID-19 (PASC, longCOVID). This past summer the Federal Reserve announced that disability rates in the U.S. have stalled their previous decline, and longCOVID has already been implicated in limiting work capacity at a national level (2). Our group previously reported that longCOVID carries significant social and economic burdens at 1 year post infection (3). In line with numerous studies describing detrimental changes in cognition, mood, behavior as predominant symptoms of longCOVID (4–10), we propose that, of the burden of longCOVID that we previously reported, brain fog in longCOVID (often known by patients as “longCOVID brain fog” (BF)) is a major contributor.

The factors that associate with BF risk longitudinally, remain unclear. Initially, many studies proposed that the degree of cognitive change associated with hospitalization severity – through the mechanism of profound nonspecific hypoxia-related damage of critical illness, neurovascular bundle compromise through thromboembolism, and acute on chronic inflammatory changes (11–17). Growing human and animal neuropathological evidence suggests that mechanisms specific to SARS-CoV-2 may contribute to long-term brain pathology (18), especially as it triggers neuroinflammation (19, 20). Neuroinflammation, without viral invasion of the CNS, may also trigger decreased hippocampal neurogenesis (21) and other anatomical effects on cognitive zones. LongCOVID also may exacerbate pre-existing brain changes (22), such as those induced by preexisting cognitive change, vascular/endothelial disease, traumatic brain injury, etc. These findings require epidemiologists to revisit the associations between long-term cognitive decline and acute COVID severity. To understand long-term implications on national workforce disability, we must further understand the risk factors associated with the cognitive symptom of brain fog and implications for neurodegenerative disease. Here we study the prevalence, risk factors, and physical and cognitive disability associated with this sometimes persistent cognitive condition, one that harbingers pathological aging and other long-term cognitive changes.

Because there are unique neuropathologic consequences in SARS-CoV-2 infection beyond typical respiratory illness, we hypothesized that severity of acute COVID-19 would not predict severity of BF. 1 year after COVID-19 infection, we aimed to understand the prevalence of BF and to clarify the factors associated with BF, including initial COVID severity (discharged from emergency room, hospitalized with/without intubation), age, sex, other medical comorbidities, factors associated with inflammation (medication, blood inflammatory markers), as well as functional capacity (activity/employment limitation and social isolation).

## Methods

Details regarding study eligibility criteria, design, procedures and materials regarding the permission, medical record utilization and survey materials at Weill Cornell Medicine have previously been described (3). Our study sample (*n* = 530) was the subset of living patients who were successfully contacted by telephone for one-year follow-up after ED visit and/or hospital admission for PCR-confirmed acute COVID-19 and responded to the question, “At this time, do you still experience any symptoms that you think are due to COVID?”

Presence of BF was determined if a patient answered in the affirmative at least once to the self-report items “brain fog (slow thinking)” within the question, “Please tell me if you have had any of the following symptoms in the past week: not at all, once, a few times, or several times.” The definition of “brain fog” is not yet formally established in the literature; patients use it to describe a confluence of negative cognitive changes including executive, attention and memory decline. As a separate long COVID symptom, patients were also asked if they had “trouble concentrating on things like reading, TV shows, or conversations” (see Table 1A, Table 1B). Other cognitive tasks were not specifically queried. Other symptoms surveyed were loss of smell/taste, numbness or weakness in extremities, chest tightness or chest pain, cough, shortness of breath, and struggle to get sleep or stay asleep.

**Table 1A:** ED visits and new comorbidities since initial COVID-19 discharge, and other PASC symptoms experienced in the past week, in respondents with and without brain fog 1  year after COVID-19 infection.

Characteristic	*N*	Overall	Experienced any brain fog		*p*-value
			No, *N* = 361	Yes, *N* = 169	
ED visits					
Any ED visits since discharge	529	126 (24%)	80 (22%)	46 (27%)	0.2
Number of ED visits since d/c	125	1.00 (1.00, 2.00)	1.00 (1.00, 2.00)	1.00 (1.00, 2.00)	0.5
Any admissions since d/c	125	78 (15%)	49 (14%)	29 (17%)	0.3
New comorbidities					
Hypertension	530	18 (3.4%)	13 (3.6%)	5 (3.0%)	0.7
Diabetes	530	10 (1.9%)	7 (1.9%)	3 (1.8%)	>0.9
Heart failure	530	6 (1.1%)	4 (1.1%)	2 (1.2%)	>0.9
Other symptoms					
Loss of smell/taste	530	40 (7.5%)	19 (5.3%)	21 (12%)	**0.004**
Numbness or weakness in extremities	530	161 (30%)	79 (22%)	82 (49%)	**<0.001**
Chest tightness or chest pain	530	59 (11%)	24 (6.6%)	35 (21%)	**<0.001**
Cough	530	117 (22%)	58 (16%)	59 (35%)	**<0.001**
Shortness of breath	530	142 (27%)	64 (18%)	78 (46%)	**<0.001**
Trouble concentrating	530	141 (27%)	28 (7.8%)	113 (67%)	**<0.001**
Struggled to get to sleep or stay asleep	530	210 (40%)	103 (29%)	107 (63%)	**<0.001**

Statistics presented are median (IQR); *n* (%), percentages are calculated with denominator as all of those who responded.

*p*-value are from the following tests as appropriate: Wilcoxon rank sum test; Fisher’s exact test; Pearson’s Chi-squared test.

Bold values indicate statistical significance.

**Table 1B:** Models assessing any brain fog as outcome in those who had any respiratory symptoms (chest tightness or chest pain, cough, or shortness of breath) compared to those who did not (referencing respiratory symptoms in Table 1A).

Models (Complete case analysis)	Any respiratory symptoms	RR^a^	95% CI	*p*-value
Unadjusted model, *N* = 530	No	–	–	–
	Yes	2.28	1.77, 2.95	<0.001
Adj. model^b^, *N* = 451	No	–	–	–
	Yes	1.54	1.13, 2.09	0.006

a Modified Poisson with Robust Standard Errors.

b Adjusted by sex, age, race, social isolation score, general health at follow-up, general health compared to 1 year ago, job change, ability to walk one block, diabetes, hypertension, CAD, BMI, CVA, smoking status, renal disease, COVID-19 severity.

Patients were also queried about new medical diagnoses and emergency department (ED) utilization in the past year, as well as current activity level, employment status change, and social network change (Lubben Score). Additionally, the original analysis abstracted data from the electronic medical records on other medical comorbidities, particularly those established as acute-COVID severity risk factors (cardiopulmonary disease, obesity, stroke, heart failure, diabetes, coronary artery disease, hypertension, current tobacco use (smoking or vaping), HIV (as it relates to immunosuppression), active cancer, liver disease, renal disease, any transplant and pregnancy) (23), factors associated with acute severity and inflammation (common acute COVID-19 medications, blood inflammatory markers mainly drawn only in admitted patients), and severity of acute COVID-19 hospitalization level. This final variable was distributed across three categories: (1) participants discharged from the ED (not hospitalized), (2) hospitalized but not intubated, and (3) hospitalized and intubated.

### Data analysis

Bivariate associations were tested using Wilcoxon rank sum test for continuous variables, Pearson’s Chi-squared test for categorical variables, and Fisher’s exact test for categorical variables with low counts. Further analyses were used to understand how BF relates to ED utilization in the past year and the presence of any respiratory symptoms (chest tightness or chest pain, cough, or shortness of breath). Risk of returning to ED for people who reported brain fog versus those who did not was assessed using a Kaplan–Meier curve and Cox proportional hazards model. To test if patients with respiratory symptoms had higher risk of also having brain fog, we used a modified Poisson model with robust standard errors adjusting for potential confounders. All analyses were completed using R 4.1.0.

## Results

The 530 respondents (median time between hospital presentation and survey 332 days [IQR 325–344]) had mean age 60 ± 11 years, 44.5% were female and 70.8% were non-White. 60.4% had cardiopulmonary comorbidities (no significant difference between groups). One year after acute COVID-19 hospitalization, we found that 31.9% of respondents (*n* = 169) experienced BF at least once in the past 7 days before the survey. 3.8% of respondents (*n* = 20) identified BF as their most bothersome symptom.

Acute COVID-19 hospitalization severity was not associated with presence of BF at year 1 (ED only 14.2% with BF vs. 13.6% without BF, hospitalization without mechanical ventilation 67.5% vs. 72.6%, hospitalization with intubation 18.3% vs. 13.9%, *p* = 0.37) The use of three common inpatient medications for severe acute COVID did not associate with the experience of brain fog 1 year later. Neither did any of five inflammatory marker levels associate with the experience of brain fog 1 year later (Table 2). Age was similar across groups (60 vs. 61 years old, *p* = 0.92). Female sex was associated with BF vs. no BF at 1 year (53.8% of those responding “yes” to BF were women vs. 40.2% of those responding “no” to BF were women, *p* < 0.003). New medical diagnoses over the ensuing year (hypertension, diabetes, heart failure) were low and did not differ between those who did and did not report BF (Table 3A).

**Table 2:** In-hospital drugs and labs for admitted COVID-19 patients who reported 1  year later that they were experiencing any brain fog within the past week.

Characteristic	*N*	Overall, *N* = 457	Experienced any brain fog		*p*-value^a^
			No, *N* = 312	Yes, *N* = 145	
In-hospital drugs during initial COVID-19 infection					
Steroids	456	90 (20%)	59 (19%)	31 (22%)	0.5
IL-6 inhibitors^c^	456	20 (4.4%)	14 (4.5%)	6 (4.2%)	>0.9
Labs (max value during hospitalization)^b^					
C-Reactive Protein (mg/dL)	351	13 (5, 25)	13 (5, 25)	13 (5, 23)	0.8
Erythrocyte sedimentation rate (mm/h)	309	87 (56, 119)	86 (57, 121)	90 (56, 114)	0.7
Ferritin (ng/mL)	348	884 (418, 1,631)	872 (438, 1,572)	908 (321, 1,745)	>0.9
Lactate dehydrogenase (U/L)	381	432 (333, 590)	420 (335, 583)	456 (318, 643)	0.5
Platelet (10^9^/L)	449	310 (217, 442)	307 (215, 447)	316 (224, 442)	0.6

a Wilcoxon rank sum test; Fisher’s exact test; Pearson’s Chi-squared test.

b Statistics presented are median (IQR); *n* (%).

c Tocilizumab and sarilumab.

457/530 were admitted. IL6 was not assessed for these patients.

N next to the column titles is the number of admitted patients in that group.

Column “*N*” contains the number of patients who received the lab work.

Patients were hospitalized or presented to the Emergency Department (ED) with confirmed acute COVID-19 between March 3 and May 15, 2020.

**Table 3A:** Comparing demographics, comorbidities, physical function, social isolation, perceived and employment statuses, 1  year after COVID-19 infection, in respondents with and without brain fog 1  year after COVID-19 infection.

Characteristic	Overall, *N* = 530^1^	Experienced Any Brain Fog		*p*-value^2^
		No, *N* = 361^1^	Yes, *N* = 169^1^	
Age (IQR)	60 (49, 70)	61 (48, 70)	60 (49, 70)	0.92
Race/Ethnicity				0.28
White	155 (29.2%)	108 (29.9%)	47 (27.8%)	
Black	62 (11.7%)	41 (11.4%)	21 (12.4%)	
Asian	79 (14.9%)	48 (13.3%)	31 (18.3%)	
Hispanic or Latino or Spanish origin	132 (24.9%)	87 (24.1%)	45 (26.6%)	
Other or missing	102 (19.2%)	77 (21.3%)	25 (14.8%)	
Sex				**0.003**
Female	236 (44.5%)	145 (40.2%)	91 (53.8%)	
Obesity (BMI >30)	158 (31.5%)	102 (29.7%)	56 (35.2%)	0.22
Unknown	28	18	10	
Current tobacco use (smoking or vaping)	18 (3.4%)	14 (3.9%)	4 (2.4%)	0.37
Comorbidity Group				0.48
Cardiopulmonary	320 (60.4%)	213 (59.0%)	107 (63.3%)	
Non-cardiopulmonary	90 (17.0%)	66 (18.3%)	24 (14.2%)	
None	120 (22.6%)	82 (22.7%)	38 (22.5%)	
Diabetes	146 (27.5%)	103 (28.5%)	43 (25.4%)	0.46
Stroke or Cerebrovascular Disease	28 (5.3%)	18 (5.0%)	10 (5.9%)	0.66
Heart Failure	23 (4.3%)	15 (4.2%)	8 (4.7%)	0.76
Coronary Artery Disease (CAD)	52 (9.8%)	38 (10.5%)	14 (8.3%)	0.42
Hypertension	266 (50.2%)	179 (49.6%)	87 (51.5%)	0.68
HIV	10 (1.9%)	8 (2.2%)	2 (1.2%)	0.51
Active cancer	15 (2.8%)	11 (3.0%)	4 (2.4%)	0.78
Liver disease (cirrhosis or Hepatitis)	13 (2.5%)	9 (2.5%)	4 (2.4%)	>0.99
Renal Disease (CKD or ESRD)	44 (8.3%)	31 (8.6%)	13 (7.7%)	0.73
Pregnancy	15 (2.8%)	13 (3.6%)	2 (1.2%)	0.16
Any transplant	20 (3.8%)	14 (3.9%)	6 (3.6%)	0.85
Severity				0.37
ED only	73 (13.8%)	49 (13.6%)	24 (14.2%)	
Mechanical Ventilation	81 (15.3%)	50 (13.9%)	31 (18.3%)	
Non-Mechanical Ventilation	376 (70.9%)	262 (72.6%)	114 (67.5%)	

1 Median (IQR); n (%).

2 Wilcoxon rank sum test; Pearson’s Chi-squared test; Fisher’s exact test.

Bold values indicate statistical significance.

Insomnia (40%) was the only symptom more commonly reported than brain fog; pulmonary symptoms were each less common. Sleep disturbance, pulmonary symptoms and weakness associated with report of brain fog (*p* < 0.0001); dysosmia/dysgeusia showed significant association (*p* < 0.004) but was less common in both BF (12%) and non-brain fog (5%) patients. Patients with longCOVID pulmonary symptoms had 54% more risk of having BF compared to those who had no respiratory symptoms, *p* = 0.006 (Table 1A, Table 1B). Interestingly, 66.9% of patients reporting brain fog also reported trouble concentrating, while 8.8% of those without brain fog had trouble concentrating (*p* < 0.0001) (Table 1A, Table 1B). Of those who responded “yes” to the question about brain fog, 80.1% also answered “yes” to the question about concentration (Table 1A, Table 1B).

**Figure 1:**
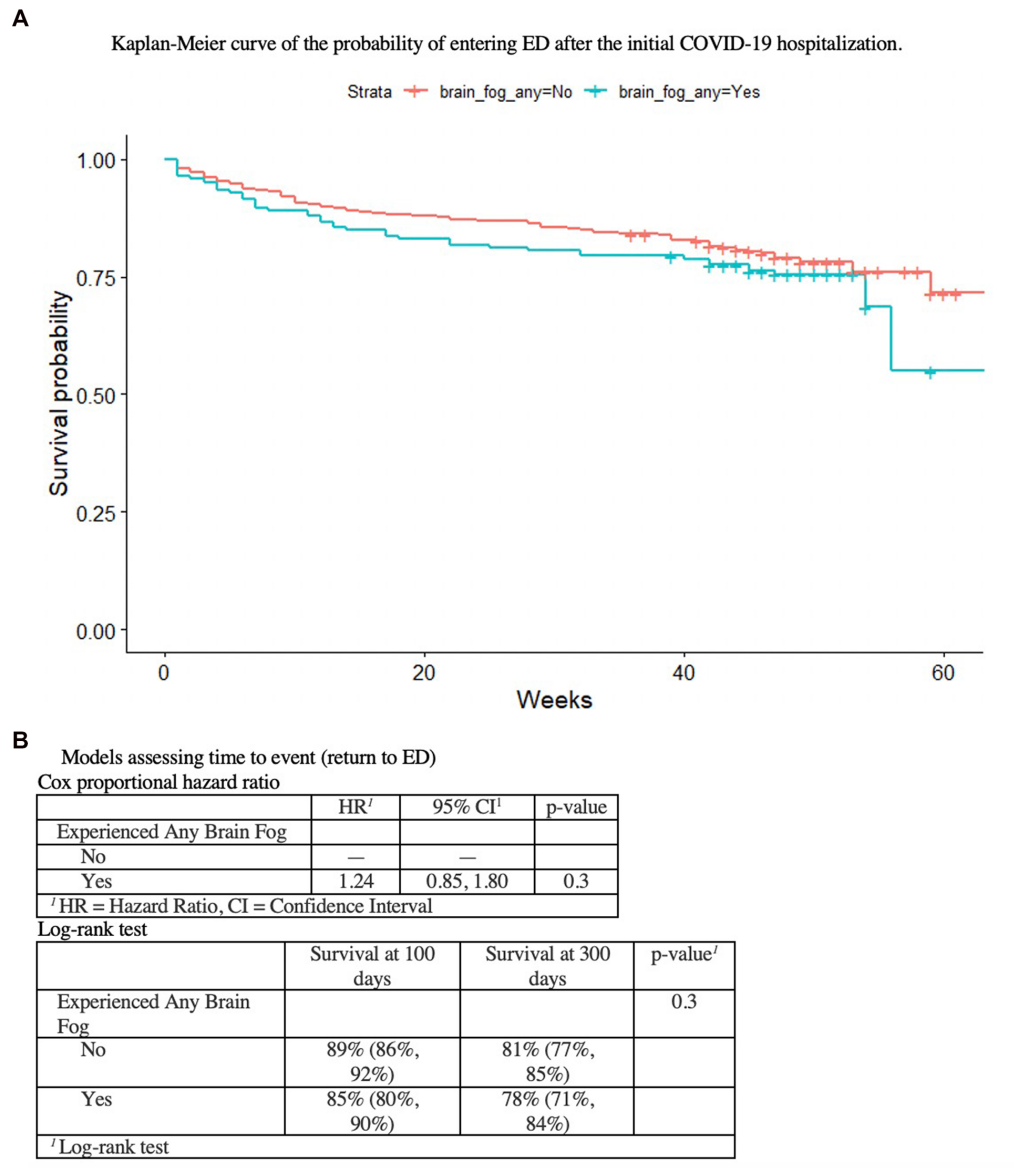
As shown in **(A)** the Kaplan–Meier curve of the probability of entering ED after the initial COVID-19 hospitalization, **(B)** the Cox proportional hazard ratio and the Log-rank test, there is no association between initial COVID-19 hospitalzation or BF at 1  year, and ED utilization.

Patients who reported BF at 1 year had various physical activity limitations (*p* < 0.001), more disability/leave (11% vs. 3%, *p* < 0.0001), worsened perceived health since acute COVID-19 (66% vs. 30%, p < 0.001) and more social isolation (40% vs. 29%, *p* < 0.02), than those who did not have BF, despite no differences in comorbid medical conditions, age, or race/ethnicity (Table 3B). There were no differences in emergency room and hospitalization utilization over the year (Table 1A, Table 1B); patients reporting BF at year 1 were no more likely to return to the ED over the course of the year (Figure 1).

**Table 3B:** Comparing physical function, social isolation, perceived and employment statuses, 1  year after COVID-19 infection, in respondents with and without brain fog 1  year after COVID-19 infection.

Difficulty with…				
Vigorous activities, such as running, lifting heavy objects or strenuous sports	332 (62.6%)	187 (51.8%)	145 (85.8%)	**<0.001**
Moderate activities, such as moving a table, pushing a vacuum cleaner, bowling, or playing golf	187 (35.3%)	93 (25.8%)	94 (55.6%)	**<0.001**
Lifting or carrying groceries	192 (36.2%)	95 (26.3%)	97 (57.4%)	**<0.001**
Climbing several flights of stairs	287 (54.2%)	158 (43.8%)	129 (76.3%)	**<0.001**
Climbing one flight of stairs	188 (35.5%)	93 (25.8%)	95 (56.2%)	**<0.001**
Bending, kneeling, or stooping	202 (38.1%)	107 (29.6%)	95 (56.2%)	**<0.001**
Walking more than a mile	242 (45.7%)	129 (35.7%)	113 (66.9%)	**<0.001**
Walking several blocks	191 (36.0%)	99 (27.4%)	92 (54.4%)	**<0.001**
Walking one block	117 (22.1%)	63 (17.5%)	54 (32.0%)	**<0.001**
In general, would you say your health is?				**<0.001**
Good to Excellent	351 (68.3%)	269 (77.3%)	82 (49.4%)	
Fair to Poor	163 (31.7%)	79 (22.7%)	84 (50.6%)	
Unknown	16	13	3	
Compared to 1 year ago, how would you rate your health in general now?				**<0.001**
Not worse	300 (58.5%)	244 (70.3%)	56 (33.7%)	
Worse	213 (41.5%)	103 (29.7%)	110 (66.3%)	
Unknown	17	14	3	
Social Isolation (Lubben Score < 14)	156 (32.6%)	94 (29.1%)	62 (39.7%)	**0.020**
Unknown	51	38	13	
Did your employment status change since March?	113 (22.0%)	67 (19.3%)	46 (27.7%)	**0.030**
Unknown	16	13	3	
Do you believe your employment change was due to COVID?	95 (84.8%)	55 (83.3%)	40 (87.0%)	0.60
Unknown	418	295	123	
On disability or sick/other leave from work	28 (5.5%)	10 (2.9%)	18 (10.8%)	**<0.0001**
Unknown	18	15	3	

^1^Median (IQR); *n* (%).

^2^Wilcoxon rank sum test; Pearson’s Chi-squared test; Fisher’s exact test.

Bold values indicate statistical significance.

## Discussion

Our cohort study design allows us to report an overall brain fog prevalence of 31% at 1 year; many other studies have used small convenience samples from single clinics that do not allow for extrapolation of prevalence of disease to a larger sample (24, 25). While the pandemic wears on and continues to cause cognitive debility, hospitalization rates have decreased since the initial 2020 wave, and policymakers are tasked with allocating resources to post-COVID brain fog. Our most important finding is that COVID-19 severity was not a predictive risk factor of brain fog prevalence, one year after acute COVID-19 infection.

Regardless of whether a patient was discharged from the ED or intubated, or whether a patient received steroids or monoclonal antibody medications then reserved for severe cases, they reported rates of brain fog at year 1 that were not significantly different. Acute COVID-19 systemic inflammatory markers also did not correlate with brain fog 1 year later. This suggests that resources must continue to be allocated for BF, even if the acute COVID-19 illness was only mild. Our study adds to evidence that highlights the importance of the study of BF risk and implications for those who did not require hospitalization. This continues to apply in the post-vaccine era as SARS-CoV-2 vaccines do not prevent infection and vaccine efficacy and penetration fluctuates with the mutating strains of SARS-CoV-2.

Furthermore, there were no significant differences in age or in preexisting comorbidities between those who reported brain fog and those who did not. In other words, preexisting conditions (most importantly, cardiopulmonary comorbidities) typically used to triage acute COVID-19 patients were not risk factors for the longCOVID symptom of BF.

Our secondary outcome was that BF 1 year after COVID-19 infection is associated with limitations in physical function, social connectedness and employment, independent of acute COVID comorbidities. Policymakers should consider BF alone as an important factor determining patients’ function, and encourage support including work accommodations, medication and non-medication based treatments (cognitive and occupational therapy).

Neither was there was a significant difference in the prevalence of brain fog between races/ethnicities (after accounting for missing data).

Out of those participants who reported any BF, there was a significant difference in sex: women reported BF significantly more often than did men. This is in line with prior reports of sex differences in longCOVID (26), and may be explained by differences in endothelial physiology in women (27) – endothelial function and blood–brain barrier dysfunction is likely an initial step in CNS longCOVID (19).

Associated longCOVID symptoms (sleep disturbance, shortness of breath, weakness, dysosmia/dysgeusia) were expected, and that patients with longCOVID respiratory symptoms were 54% more likely to have BF reflects several postulated systemic and cerebral overlapping longCOVID pathophysiologies (e.g., endothelial vascular dysfunction, persistent hypoxemia). It is interesting that insomnia was more commonly reported than brain fog. Also, most patients, but not all, experience both brain fog and trouble concentrating concomitantly. However, a minority does not, and it is unclear if this represents a different pathology. This speaks to the importance of broadly capturing cortical and subcortical functions and broad neuropathology related to brain fog (28, 29) – only two of which are the executive tasks of slowed processing and poor concentration (others reported in our clinic include confused processing, foggy feeling in the head, difficulty with ordinary tasks, short-term memory loss, learning difficulty, etc). This also suggests the importance of other mechanisms less heavily investigated (which may be impacted by sleep quality (30, 31)), such as CNS clearance (32) and perfusion/metabolism (33, 34).

## Limitations

The cross-sectional nature of this cohort does not allow for causality, only associations, and is meant to establish the need for more rigorous retrospective case-cohort and prospective studies. Factors affecting BF over the year were not fully captured (medications and medical treatment, socioeconomic status, etc); we may be underestimating total prevalence of BF, as some patients likely succeeded in resolving their disease (with varying degrees of medical intervention) prior to the survey. Prevalence may also be underreported due to the fluctuating nature of longCOVID cognitive symptoms. Age was not a risk factor but it is notable that our population was middle-to-older age, and is less representative of the many younger adults who are suffering longCOVID. Other highly prevalent longCOVID symptoms including fatigue and mood changes (24) were not queried. Utilization rates were obtained from patient questionnaires rather than the chart; there are many local hospital systems and limiting our analysis to just those in our medical chart would have been an inaccurate representation. We do not know whether treatment with key medications, such as steroids, antivirals, monoclonal antibodies, and cognitive medications, was given in the outpatient period after hospitalization. Given that the case and control groups were both patients who were seen at one hospital center over one period of time, we make the assumption that these rates were similar, but they could be a confounder. The study most directly reflects effects of the original alpha SARS-CoV-2 variant. However, given that severity of acute COVID-19 here is not a risk factor for BF, this finding is likely still applicable to milder SARS-CoV-2 strains in circulation today, such as omicron. Most importantly, this sample represents only those who sought hospital opinion and were either hospitalized or discharged from the ED. While we do extrapolate a trend from our cohort of less-to-more severe acute COVID-19 infection, we do not know whether even-less-severe acute COVID-19 infections (for which patients did not choose to seek hospital care), would have followed the same trend. More recent studies and our clinical experience suggest that variants of post-COVID brain fog may be reliant upon heterogeneous underlying risk factors or host factors (perhaps also some acute COVID-19 risk factors such as type 2 diabetes) (35) – in future studies it will be important to also capture certain emerging risk factors such as underlying cognitive impairment or dementia, previous viral infections, dysautonomia immune-activation/senence, endothelial disease, and atopy (Supplementary material 1).

## Conclusions and clinical implications

By acknowledging the high prevalence of BF in the population over time, we can better position our clinical efforts to serve our community in the outpatient setting. Our findings warn that focusing on severity, preexisting acute COVID-19 comorbidities, and age may not accurately predict existence or severity of brain fog. Rather, female sex and overlapping longCOVID respiratory symptoms were risk factors. Activity limitation and social isolation emerged as factors associated with BF independent of acute COVID-19 comorbidities. The economic burden of “brain fog” to patients and the society as a whole is mounting. We propose targeted brain fog investigations to curb this worrisome trend in national disability, and to better understand its long-term implications for further persistent and perhaps progression of cognitive disease. BF is an important long-term sequelae of COVID-19, regardless of initial severity, and independently requires policymaker consideration and scientific research investment.

## Data availability statement

The raw data supporting the conclusions of this article will be made available by the authors, without undue reservation.

## Ethics statement

The studies involving human participants were reviewed and approved by Institutional Review Board, Weill Cornell Medicine. The patients/participants provided their written informed consent to participate in this study.

## Author contributions

All authors listed have made a substantial, direct, and intellectual contribution to the work and approved it for publication.

## Funding

This study was supported by the NIH R01AG068398 (GC) NIH RF1AG057570 MdeL NIH R56 AG058913 MdeL Feil Family Clinical Scholar in Neurology II Award (AN) NIH/NINDS NeuroNext Fellowship 5U24NS107168 (AN).

## Conflict of interest

The authors declare that the research was conducted in the absence of any commercial or financial relationships that could be construed as a potential conflict of interest.

## Publisher’s note

All claims expressed in this article are solely those of the authors and do not necessarily represent those of their affiliated organizations, or those of the publisher, the editors and the reviewers. Any product that may be evaluated in this article, or claim that may be made by its manufacturer, is not guaranteed or endorsed by the publisher.
