# Supplementary figures and images for: Brain fog in long COVID limits function and health status, independently of hospital severity and preexisting conditions
Source: Front Neurol. 2023 May 11;14:1150096. doi: 10.3389/fneur.2023.1150096 (PMC10213727; doi:10.3389/fneur.2023.1150096)

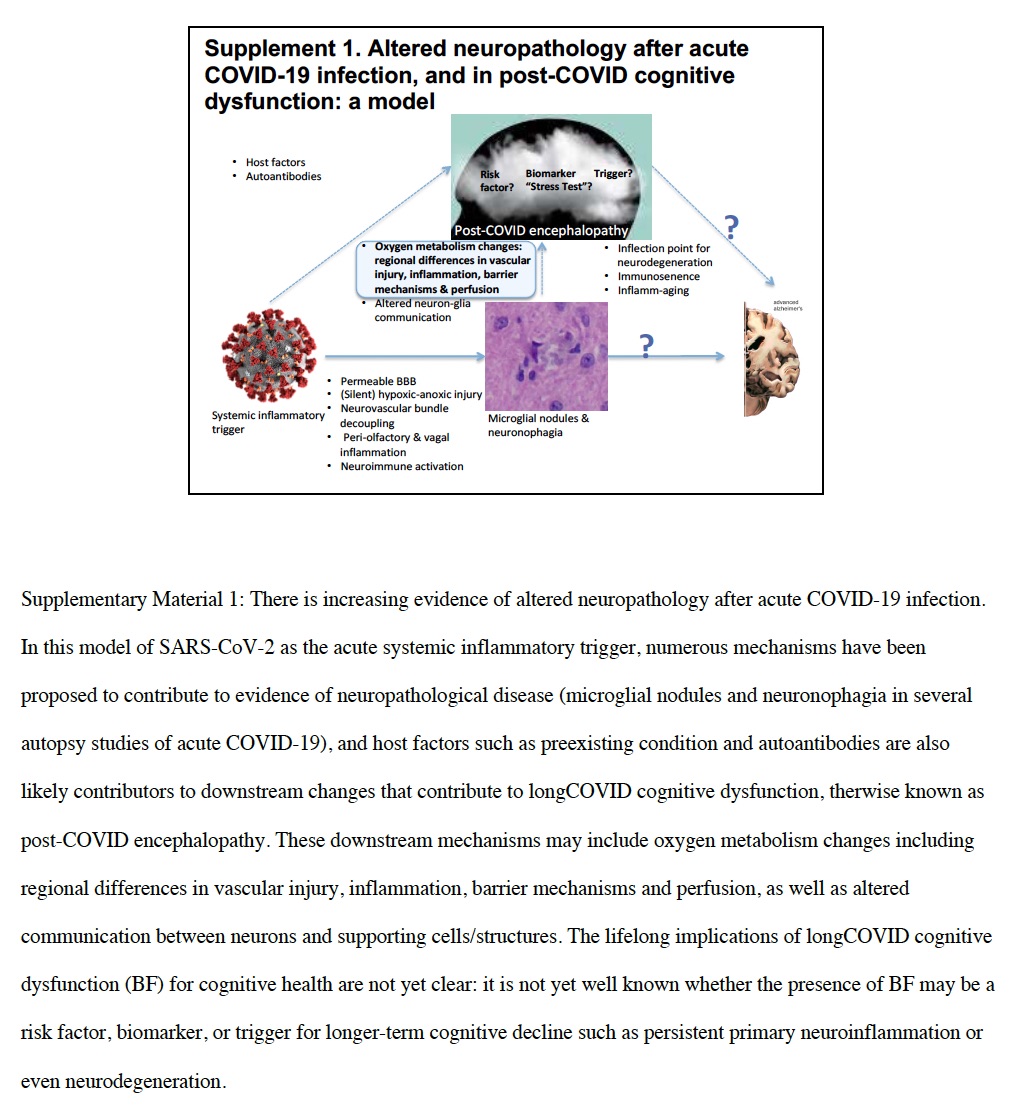

Supplement: Supplementary file 1 [file Image_1.JPEG]
